# Supplementary material for: Wealth and obesity in pre-adolescents and their guardians: A first step in explaining non-communicable disease-related behaviour in two areas of Nairobi City County
Source: PLOS Glob Public Health. 2023 Feb 28;3(2):e0000331. doi: 10.1371/journal.pgph.0000331 (PMC10021148; doi:10.1371/journal.pgph.0000331)
Supplement: S1 Text — (DOC) [file pgph.0000331.s002.doc]

**S1_Text: STUDY PROTOCOL**

**Wealth and obesity in guardians and pre-adolescents: a first step in a study explaining non-communicable disease-related behaviour in two areas of Nairobi City Country**

Ochola S^1^, Wachira LJ ^2^, Owino GE^3^, Anono EL^1^, Kanerva N ^4^, Walsh H^4^, Okoth V^5^, Erkkola M^4^, Swindell N^6^, Stratton G^6^, Fogelholm M^4^, Onywera V^2^.

^1^Department of Food, Nutrition and Dietetics, Kenyatta University, Nairobi, Kenya

^2^Department of Physical Education, Exercise and Sport Science, Kenyatta University, Nairobi, Kenya

^3^Department of Sociology, Gender and Development Studies, Kenyatta University, Kenya

^4^Department of Food and Nutrition, 00014 University of Helsinki, Finland

^5^Department of Environmental Science, Kenyatta University, Nairobi, Kenya.

^6^Applied Sports Technology, Exercise and Medicine Research Centre. Faculty of Science and Engineering, Swansea University, UK

**A detailed description of design, methods and practical experiences**

**Study Design**

The design of the whole study is shown in S1 Fig 1. The two methodological approaches (quantitative and qualitative) are used to get a comprehensive picture of non-communicable disease-related behavior in Nairobi City County, Kenya, in the context of family and poverty.


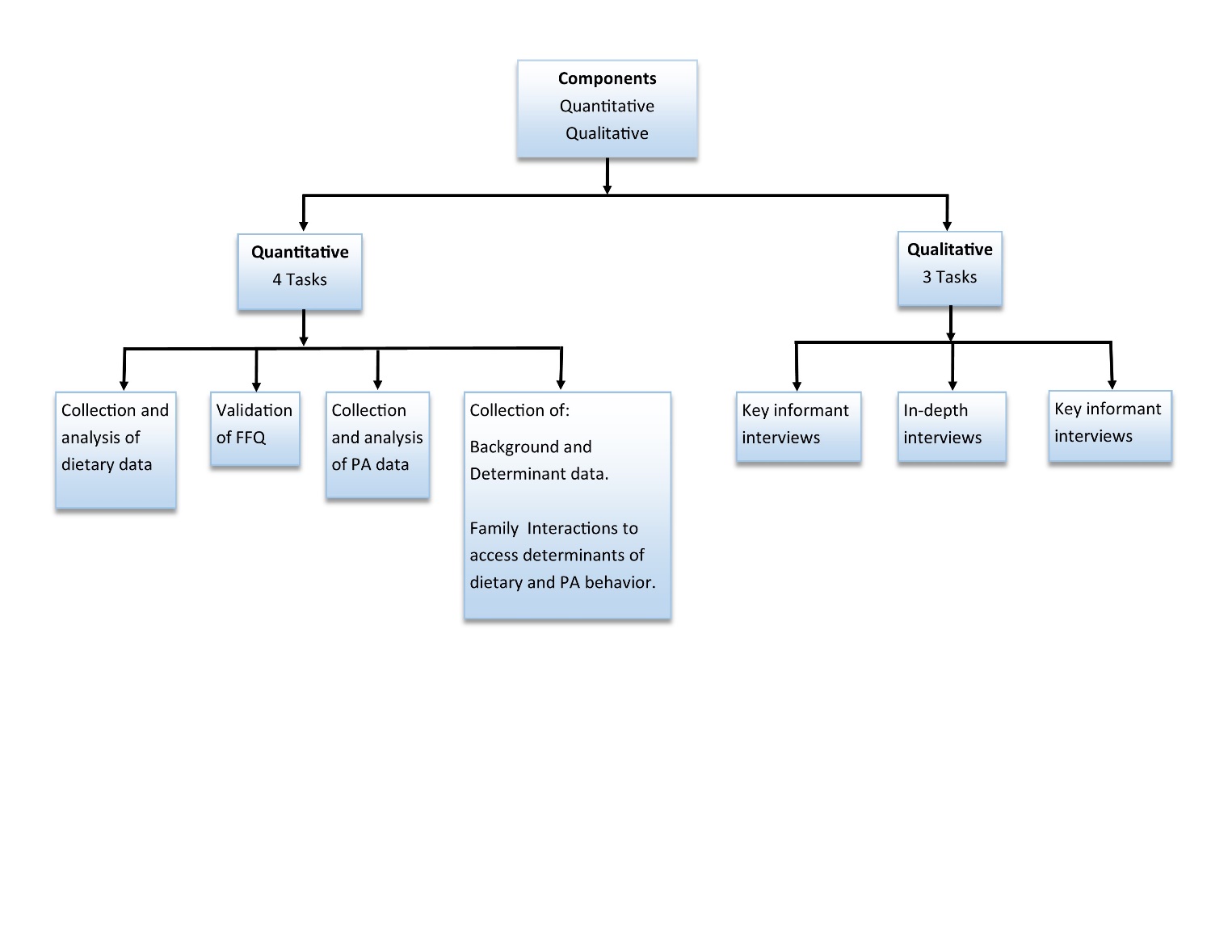


SI_Fig1: Flow chart showing study design and organization

**Study setting**

The study was carried out in Embakasi central sub-county (Kayole South Ward) and Langata sub-county (Nairobi West Ward) within Nairobi County (S1 Fig 2). The two areas are located about 10 km from each other. Kayole South Ward in Embakasi consists partly of informal settlements, whereas Nairobi West Ward in Langata can be characterized as a middle, even upper-middle SES area.

**
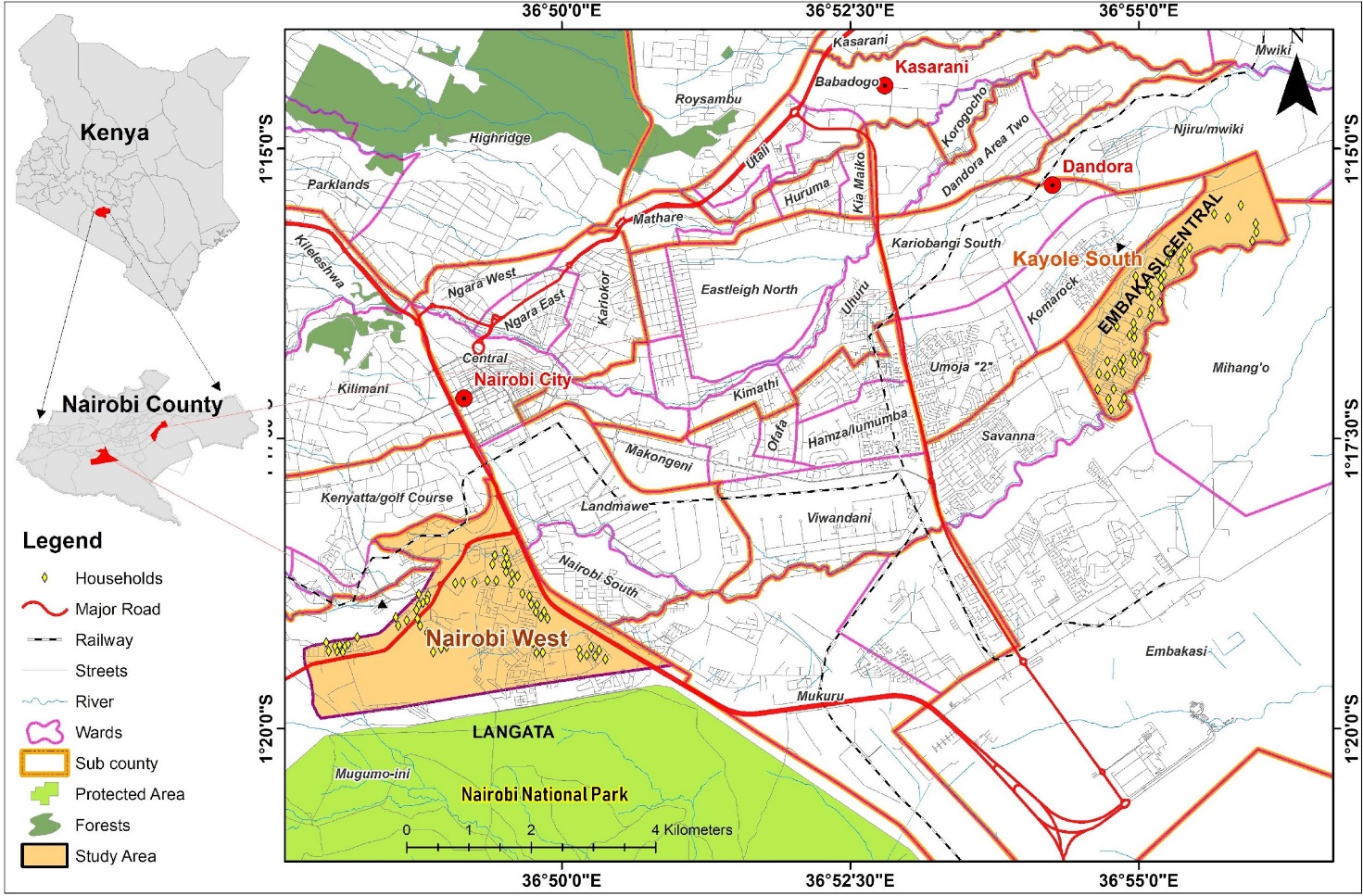
**

S1_Fig2: The two study areas are shown on the map of Nairobi.

RCMRD, G. (2020, 5 15). *RCMRD GeoPortal*. Retrieved from https://geoportal.rcmrd.org/layers/servir%3Akenya_county_boundary#more

**Quantitative study part**

Study population and sample size

The study targeted families of low or middle SES with pre-adolescents in the age range of 9-14 years and their guardian(s), residing in the selected study regions in Nairobi County. The main inclusion criteria were that the family has at least one pre-adolescent aged from 9-14 years and at least one parent or guardian available, and who have been residents of Langata or Embakasi for at least 6 months before the study. Moreover, the family had to sign an informed consent, hence be a voluntarily participant. The study included the pre-adolescent and guardian(s) (mother and/or father, if both belonged to the family and regardless of whether they were biological parents or not). If there was more than one pre-adolescent in the target age range, the participating pre-adolescent was drawn randomly. Households having 9-14 years old with documented chronic disease conditions, such as tuberculosis, impacting diet, or who had any significant illness preventing participation were excluded from participating in the study.

Sampling procedure

For the quantitative part, a multi-stage sampling technique was conducted to identify the households where data would be collected. Embakasi Central represented low SES and Langata Sub-county the middle SES. In the second stage of sampling, Kayole North Central Ward and Nairobi West Ward were purposively selected from the sub-counties respectively. These two Sub-counties are densely populated and would therefore provide an adequate sample of the target population (pre-adolescents). In the third stage, five villages were selected randomly from Kayole North Central sub-county and 12 estates from Langata Constituency. The fourth stage of sampling involved the enumeration of households with pre-adolescents in the age range 9-14 years from the selected villages and estates with the assistance of Community Health Volunteers (CHVs). In total 223 households were enumerated in Kayole South Ward and 173 households in Langata. Simple random technique was used to select 80 households from Kayole and 80 households from Langata. The sample size (70 per study side) was inflated by about 10% to cater for non-response. The final sample sizes were 72 for Kayole and 77 for Langata..

The flowchart showing the sample selection procedure is shown in Supplement 1, Figure 3. We used a mixture of purposively and randomly selected samples. The sub-counties and wards were purposively selected to allow us to test the study hypotheses. However, within these areas, the selection of specific target areas (villages or estates) and the selection of families were done by random draw.


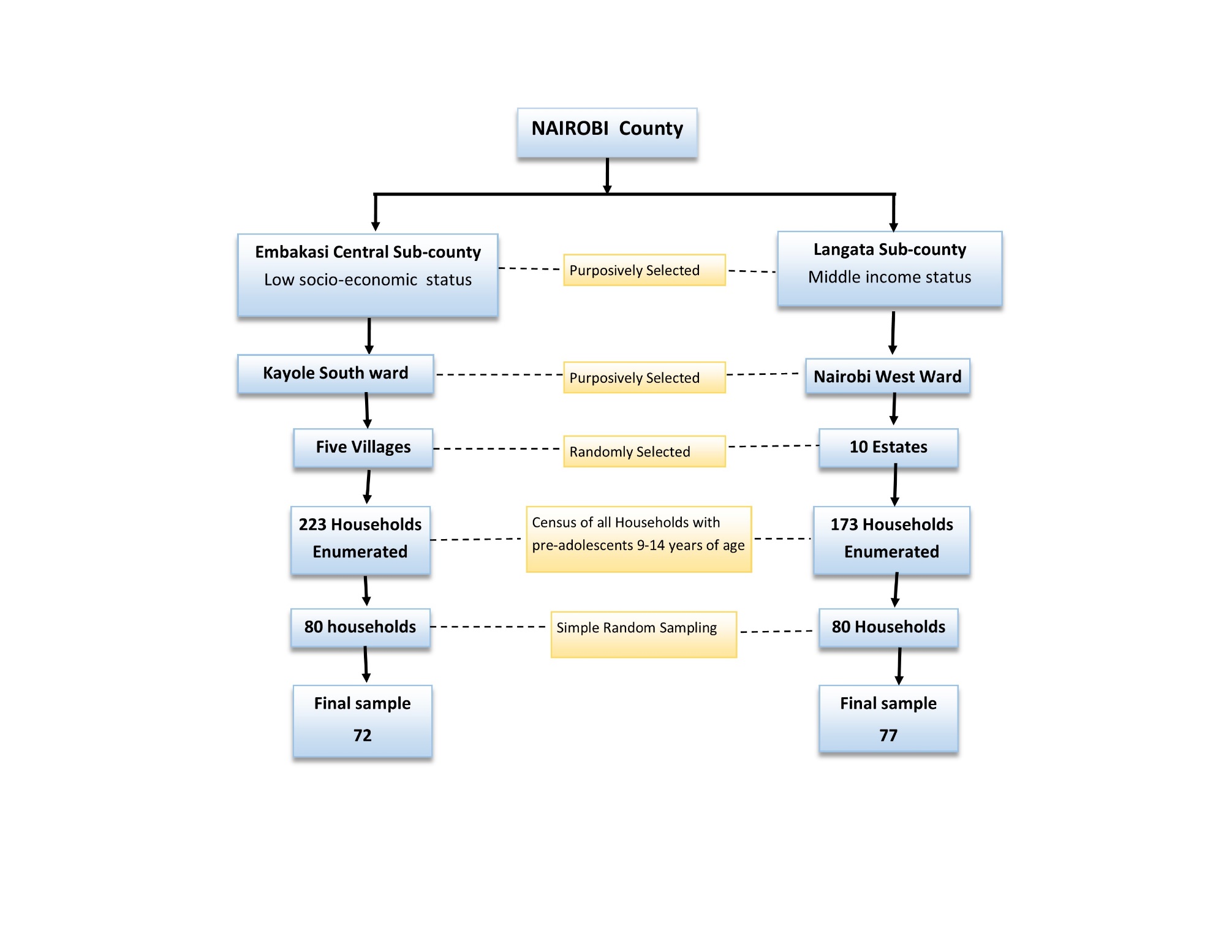


S1_Fig3: Flow chart showing the sampling procedure

Quantitative data collection and processing

To collect the quantitative data, field assistants visited the households twice approximately 8 days apart during April-June 2019. The field assistants received a one-week training to introduce them to the data collection and study protocol. Before the data collection, a brief pilot period was conducted in April 2019. The study visits were conducted based on prior appointments and conducted after school on weekdays. During weekends, the interview hours were more flexible. During the first visit, the pre-adolescents and guardians completed questionnaires on demographic and socioeconomic background, physical activity, and food consumption frequency. Further, pre-adolescents' and guardians’ anthropometric measurements were taken, and a 24-hour dietary recall interview was administered to the pre-adolescents. Besides, the participants received accelerometers. During the second visit, the accelerometers were collected, a second 24-hour recall was conducted, and the questionnaire on parenting style was administered to the guardians.

## Background data of study participants

The interviewer-administered questionnaires and qualitative interviews were in English and Kiswahili languages. The quantitative data was collected electronically by use of android phones/tablets using purposefully developed questionnaires developed on Open Data Kit (ODK) software [1], in a face-to-face interview during household visits.

A structured and validated questionnaire was used to collect demographic and socioeconomic characteristics, as well as housing conditions and ownership of assets [2]. The questionnaire included information on participants’ sex, age, marital status, education, income, occupation, the form of employment, household expenditure on various items, the materials from which the household is constructed based on the questionnaire used in the Demographic and Health Survey in Kenya [2]. This questionnaire was administered to guardians.

*Parenting practices questionnaire*

The study utilised a validated questionnaire on parenting strategies for the Eating and Activity Scale [3] to measure the influence of parenting practices on NCD-related behaviour, such as a child’s diet, physical activity, and screen-based behaviours.

Dietary data

*24-hour dietary recall*

The study adopted the use of a repeated 24-hour recall to collect detailed information on dietary intake of the pre-adolescents, which was undertaken on two non-consecutive days that included one weekday and one weekend day to minimise random error and to capture the variation between weekdays and weekend days. The 24-hour recall was undertaken by trained research assistants using a standard protocol [4]. The data collected included a description of the foods eaten; the cooking methods; brand names (e.g., for cereals, processed snacks); and the amount of food consumed which was estimated by the pre-adolescent themselves and where necessary assisted by their guardians. For accurate reporting of food intake, pre-adolescents were shown the Photographic Food Atlas for Kenyan Pre-adolescents consisting of portion sizes of common Kenyan dishes, developed for this study and pilot-tested for feasibility [5]. 24-hour recall data was checked by a nutritionist. Each food is linked with equivalent food from the Kenyan Food Composition Tables 2018 [6] for calculating nutrient intakes.

*Food Frequency Questionnaire (FFQ)*

The study adopted the use of a 7-day semi-quantitative FFQ with 174 food items for pre-adolescents and a non-quantitative version of the same FFQ for guardians. The FFQ was used to assess the usual food consumption patterns using a standard protocol. The FFQ used was based on a previously validated FFQ for adults used in Nairobi [7] and was adapted to this study by including culturally relevant foods, urban specific as well as foods commonly consumed based on market survey and pre-test of the FFQ in the study area. The food items were categorised into 12 food groups based on FAO guidelines for grouping foods [8]. Using the ODK software, the interviewer asked (one food group at a time), which foods the participant had eaten during the past week. Then, participants were asked to report how many times they had eaten these foods during the past week. The ODK allowed the participants to report the exact frequency per day or week without any prior categorisation to, e.g., “1-3 times per week”. In addition, pre-adolescents were asked to indicate their usual average portion size of the reported food items consumed during the past week by use of the Photographic Food Atlas for Kenyan Pre-adolescents (picture number and portion size code were recorded to ODK). For amorphous foods, participants were allowed to select portion sizes that were bigger, smaller, or in between the presented portion sizes (smaller than A, between A and B, between B and C, more than C) as this is more accurate than indicating fractions of a food portion [9—12]. For foods that come in distinct units such as bread, cakes, and fruits, participants were allowed to indicate how many pieces they have consumed, which resulted in a multiplication factor of the portion size (e.g., 0.5x, 1x, 2x, 3x, and 4+x). The pre-adolescents answered the questions themselves, but the guardians were allowed to help when needed.

After data collection, the reported frequencies were checked by nutritionists. For the pre-adolescents FFQ, all consumption frequencies of three or more times a day were checked, and Kenyan experts gave their opinion on which frequencies were acceptable and which were too high for each food item. The frequencies deemed too high (e.g., for sunflower oil, tea, traditional maize stiff porridge called *ugali*) were compared to consumption frequencies in the pre-adolescent's 24-hour recall and an average daily intake was calculated based on these. All adults’ consumption frequencies of three or more times per day were listed. There was no 24-hour recall data for adults, so instead absolute maximum frequencies for daily consumption frequencies were determined by the Kenyan team based on local knowledge. For calculating the daily food consumption in grams, the portion size code reported in ODK was first linked with the correct portion size weight in grams in R software. After this, the portion size weight was multiplied by the possible multiplication factor in the case of the foods served in units and then multiplied by the frequency per day. Later on, each FFQ food item was matched with an equivalent food from the 2018 Kenyan Food Composition Tables to calculate estimated daily intakes for nutrients (in grams, milligrams, and micrograms).

Physical activity data

A validated Active Transport and Physical Activity Assessment questionnaire [13] was used to assess the types of physical activities engaged in a typical week and active transportation engaged in daily. The questionnaire also sought to assess perceived barriers to physical activity among the pre-adolescents and the guardians.

In addition to the physical activity questionnaire, the study used the ActiGraph GT3X+ Accelerometers (ActiGraph LLC, Pensacola, FL) for pre-adolescents and ActiSleep+ (ActiGraph LLC, Pensacola, FL) for guardians to objectively monitor activity, including sedentary time and sleep. The accelerometer, attached to a flexible belt was instructed to be worn at the waistline for 24 hours a day on at least 8 consecutive days excluding bathing, showering, or swimming time. Oral and written instructions of use were given with the accelerometer. At least four valid days including one weekend day (≥10 h/day of waking wear time) was required for the participant’s data to be included in the analysis.

Accelerometer measurements have been previously validated among Kenyan children [14]. The data was downloaded onto a computer using the ActiLife analysis software package (Actilife software version 6.13; ActiGraph, Pensacola, FL, US), reviewed for completeness, and analysed using Kinesoft. Using an algorithm, nocturnal sleep episodes were removed from the data. For pre-adolescents, non-wear periods were defined as any sequence of at least 20 consecutive minutes of zero activity counts. For guardians, non-wear was classified as 60 minutes of consecutive zeros with the allowance of interruptions up to 2 minutes. To assess the intensity of physical activity, the cut points of Evenson (pre-adolescents) [15] and Troiano (guardians) [16] were used to define time spent on sedentary behaviour, light, moderate, and vigorous physical activity.

Anthropometric measurements

Weight and height of pre-adolescents and their guardians were measured with minimal clothing on and shoes off, using a digital electronic scale (Seca Robust 813) to the nearest 0.1 kg and a stadiometer (Seca 217) to the nearest 0.1 cm. BMI-for-age z-scores for pre-adolescents were calculated, using WHO’s growth references [17]. For adults, BMI (kg/m^2^) was calculated by dividing weight by the square of height. Waist circumference (WC) was measured, using a waist circumference tape (Edtape for body measurements) to the nearest 0.1 cm around one’s body about halfway between the bottom of the lowest rib and the top of the hip bone, roughly in line with their belly button over light clothing or skin. The Mid-Upper Arm Circumference (MUAC) of the left upper arm was measured at the mid-point between the tip of the shoulder and the tip of the elbow using a non-elastic anthropometric measuring tape (to the nearest 0.1). Each of the measurements was taken twice and an average was calculated to ensure accuracy.

Statistical analysis of quantitative data

Statistical analyses were done using SPSS (IBM SPSS Statistics version 25) and R software [18]. Household characteristics were calculated by study area and for the total population as means and standard errors for continuous variables and as counts and percentages for categorical variables.

Wealth is often measured in terms of economic status and living standards of households. However, the income, expenditure, and consumption data needed for calculating these can be challenging to measure accurately. For this reason, we chose to use the Wealth Index similarly to the Demographic and Health Surveys (DHS) [2] and World Food Programme (WFP) Surveys [19]. The index was created according to the WFP VAM Guidance Paper [20]. Principal component analysis (PCA) (SPSS command FACTOR, Method: Principal components, Varimax-rotation) was applied to combine information on asset ownership and housing characteristics. Where necessary, variables were recoded into binary variables based on the knowledge and insight of Kenyan researchers. First, the frequencies of the wealth indicators were explored in both areas (Supplement Figure 2). The indicators that existed in over 95% or less than 5% of the households were removed. These included: electricity, mobile phone, solar panel, table, sofa, and bed. The Kaiser-Meyer-Olkin (KMO) test was used to determine the sampling adequacy of data and to ensure that the data were suitable to run a Factor Analysis. KMO values between 0.8 and 1 were set to indicate adequate sampling. The correlation test printed out with the PCA was used to evaluate whether correlations were too high meaning certain variables measure the same thing. This led to removing improved drinking water, motorcycle, improved wall material, radio, and cassette or CD player. The final wealth index had KMO value of 0.871 and it explained 40% of the total variation (Supplement Tables 1-4). The index included sanitation, floor material, television, refrigerator, chair, cupboard, wall clock, microwave, DVD player, electric or gas stove, kerosene stove, bicycle, and car or truck. The wealth index was further grouped by using quantile classification.

**The qualitative part of the study**

*Sampling and Sample Size Determination*

The qualitative part of the study used the same setting, but a separate sub-sample of participants described in S1 Fig1. The main difference between the quantitative and qualitative parts of this study was that the interviewees and focus-group participants were purposively selected with the help of community health volunteers. Purposive sampling enables researchers to use their judgment to sample respondents who are best suited to provide the data needed to respond to the objectives of the study.

The study involved eight key informant interviews (KIIs) from each of the study sites making a total of 16 KIIs. Key informant interviews were conducted with the Sub-county medical officer of health and the Sub-county nutrition officer. Other key informants included a headteacher at a local primary school, the area chief, games and sports officer, a local food vendor, a community health worker, and a village elder (one each from each of the study areas). Further, thirty (30) in-depth interviews (IDI) were conducted in total with mothers/guardians from purposively selected households: 15 interviews per study site. Conventionally, the recommended sample sizes for interviews range from 15-30 as this range is considered as the point of saturation, a point beyond which additional interviews do not yield new information [21].

Additionally, a total of 12 focus group discussions (FGDs) were conducted with fathers, caregivers/mothers, and pre-adolescents. Two FGDs were conducted per site with each of these three target groups by use of an FGD guide. The recommended number of FGDs for reliable and credible qualitative results is between 4-8 FGDs. Thus our 12 FGDs meet the minimum threshold [22].

*Conducting qualitative interviews and FDGs*

We collected data through three main qualitative research methods as indicated above. The three data collection methods and the three different target groups were used to achieve source and method triangulation of findings, a key criterion for achieving credibility or validity of qualitative findings [23]. Data from different target groups and generated through different methods could be mutually reinforcing.

KIIs were conducted at the health facilities. IDIs were conducted within participants’ homes or at sites, they deemed comfortable in. For these interviews, a semi-structured interview guide was used. The interview guides for the pre-adolescents were less detailed than those for the mothers/guardians. FGDs were conducted with the help of an FGD guide containing the same themes as those used in the KIIs and IDIs. The FGDs were conducted in health facilities, locally available halls, and in the chief’s offices. The interviews and FGDs were audio-recorded and were transferred from the recorder and imported to a google drive folder opened for this research which only the study members had access to. Themes considered in the interviews and the FGDs are shown in S1, Table 1.

S1_Table 1: Thematic areas of the qualitative study

|  | **Broad Thematic area** | **Theme** | **Sub-themes** |
| --- | --- | --- | --- |
| 1 | Meanings of food, eating, overweight and obesity | Meaning of food, eating, overweight and obesity | Thoughts about:   - food and eating, - foods that are considered healthy - Foods considered unhealthy |
|  |  | Eating and nutrition habits and cultural Practices | - Foods the people eat and their sources - Frequency of eating - Priorities in the type of foods eaten - Cultural practices and beliefs associated with food and eating |
|  |  | Beliefs about health | - People considered to be health - Thoughts about people who are obese - Perceptions on the link between upsurge of NCDs and eating - People considered to be at risk of NCDs - Perceptions on the solutions to the problems associated with NCDs |
| 2 | Norms and beliefs related to physical activity | Thoughts about physical activity | Thoughts about:   - Thoughts about the meaning of physical activity - About who should be engaged in physical activity - The health benefits of physical activity - High-status people engaging in physical activity |
|  |  | Physical activity practices | - Regularity of exercise - Likelihood of engagement in physical activity - Links between physical activity and health. |
| 3 | Environmental matters |  | - Challenges of living in urban life - Solution of challenges to urban living |

Data quality was assured through supervision from the research team through daily debriefs and peer-to-peer reviews. This was also achieved through appropriate recruitment of study participants taking into account differences in the population (i.e., religion, culture, education, gender, etc.), creating rapport and a conducive environment for discussion, and appropriate techniques to enhance the gathering of relevant information.

*Qualitative analyses*

After data collection, the audio records were transcribed and coded to facilitate analysis and report writing. Each of the KIIs, IDIs, and FGDs was transcribed verbatim in the interview language (Kiswahili, Sheng, English) soon after data collection ended. The audio records were augmented by field notes taken from the field during data collection to fill in gaps where there was noise in the recording or where the interviewees were not audible. Transcribing each interview took an average of 5 hours for one hour of interview recording with the FGDs taking the longest. The transcribed files have been coded based on the main themes of the study and saved in rich text format and have been imported to MAXQDA Analytics Pro 2019 (VERBI GmbH release 18.2.0, Berlin) as two separate folders representing the two study sites. The identities of all the group participants were kept anonymous by use of identifiers instead of names for confidentiality.

*Ethical considerations*

For those participating in the qualitative part, consent forms for in-depth interviews, key informant interviews, and FGDs were collected respectively. Informed consent was also sought for the recording of the interviews and FGDs.

**Comments on the methodology**

The study used newly developed methods – the use of the Photographic Food Atlas to estimate the amount of food consumed by the pre-adolescents. The Food Atlas was specifically developed and piloted tested for use in this study because Kenya does not have any Food Atlas for this and other population groups. The majority of the studies conducted in Kenya on dietary intake rely on the use of household utensils and measures for estimation of food consumption. Secondly, the use of digital technology in the collection of dietary intake data using questionnaires on the ODK platform is unique in the field of nutritional sciences. Additionally, preliminary food nutrient data information can be calculated by linking Food Composition Tables to the Food Atlas on the ODK platform, therefore, reducing time spent on data analysis and also improves daily quality by minimizing errors in data entry.

Diet and physical activity have been studied earlier in Kenya, but with simpler methods than conducted in this study [24]. In dietary analyses, we will be able to go from nutrient intakes and consumption frequencies of single foods to quantitative estimation of total dietary patterns. In terms of physical activity, by using accelerometers, we will be able to separate different intensities, including sedentary time and sleep.

Such detailed data on lifestyle behaviour in an African context are novel and they give a unique insight into urban residential areas with varying SES. These data are urgently needed to understand behaviour and to plan health promotion interventions and programs. Moreover, the possibility to use a combination of quantitative and qualitative data is rare and it may bring new insights.

There is anecdotal evidence suggesting that the use of active transport (cycling, walking, and jogging) may be considered inappropriate for people of higher social classes. These perceptions may thus inform whether a person adopts a healthy exercise and an active transport routine or not. Evidence also suggests that the perceived ideal body size favours being overweight and obese [25]. Being overweight or obese in Africa is considered a sign of wealth, health, attractiveness, respect, and dignity, whereas lean body image may be associated with HIV or poverty. The study examined these research questions by using thematic interviews and focus group discussions. Studies conducted on these aspects but not this kind of depth, and in the context of different residential areas and SES in an LMIC setting in Kenya.

It is anticipated that the results of the present proposal can be extrapolated to other sub-Saharan African countries facing similar challenges and with comparable socioeconomic profiles and trajectories. The FFQ, modified for pre-adolescents in the present study and to be validated within the project, will provide a useful tool to be used in later studies among the same age groups in Kenya and neighbouring countries with similar dietary patterns.

**Practical lessons learned**

The researchers learnt several lessons from the implementation of this research. Government and community support, and getting approval and political buy-in are critical for the success of a field-based research project. Field research can be full of surprises and can take time and therefore the planning needs to be flexible in terms of the planned duration of the study.

The training for the data collectors was conducted over 5 days plus an additional one day for pre-testing of data collection tools. The multiple-day training for data collectors accorded them the opportunity to practice data collection skills and taking anthropometric measurements. The multidisciplinary team of data collectors, which consisted of nutrition, physical activity, sociology students provided the expertise needed for the collection of data from the various thematic aspects of this study. Data was collected by the use of digital technology – using mobile android phones and tablets on the ODK platform. This speeded up data collection and data analysis and improved quality as there was no missing data.

Our experience from this study is that the majority of the local people need more insight into the importance of scientific research. Researchers working in similar environments and circumstances should do more in terms of creating awareness of the importance of research to the study population. Often researchers only do one-day awareness sessions with the local administration leaders, but this is not adequate. The people from the low SES class are agreeable to participate in research but feel they are overburdened and do not see the effect of the research in their lives. Researchers should disseminate and give feedback to the communities where studies are conducted, explaining explicitly how the study findings will be used for the benefit of the study participants. This is a research ethical requirement, but many times not fulfilled by researchers.

We observed that those from the middle SES class were either too busy or perhaps did not value the social benefits of research, and therefore many times they did not keep the interview appointments. Moreover, the community health volunteers do not work that closely with the people from the middle and higher SES classes as they do with those from the lower-income areas. We, therefore, recommend that for middle and higher SES classes in an LMIC context, a plan for a longer data collection period as well as education on why research is important.

There were issues with the wearing of accelerometers for the collection of data on physical activity. Some teachers did not approve of students wearing these gadgets. The research team had to go to the schools and inform and seek permission for the students to wear them while in school. This was an oversight on the part of the research team that originally only sought approval from the household heads. Secondly, it is important to take into account the family structure. It is important to get consent from both husband and wife for participation in a study. Some husbands were not consulted and only discovered when they discovered their wives wearing the accelerometers in bed. Some fathers were also not amused to discover their children wearing the accelerometers.

The FFQ for pre-adolescents was too long and contained too many foods, some consumed by very few participants or not at all. There was a tendency for some of the pre-adolescents to report that they consumed almost all the foods while some of the pre-adolescents needed assistance from the guardians to respond to the FFQ because of recall bias, level of understanding, and literacy. The research team will review which food items were consumed by very few and remove them in the future to shorten the FFQ. We already know that 21 out of the 174 food items in the Food Atlas were not consumed by anybody and thus will be excluded also from the Food Atlas. From our experience, we also recommend that researchers should do a market survey to be acquainted with the relevant foods for the context in which the research is to be done since seasonality is an important aspect affecting dietary patterns.

For FGDs with pre-adolescents, it is important to ensure that the children are relaxed and understand their role in the discussion. FGDs should be kept short and the guiding questions simple and clear.

**Societal Implications of the research findings**

Research findings will provide useful information to researchers and stakeholders such as the Ministry of Health for promoting healthy diet and physical activity behaviour to ensure optimum pre-adolescent growth and development. Further, it will inform policy decisions on the appropriate strategies to improve pre-adolescent health behaviour through dietary and physical activity interventions addressing behaviour modification.

The findings of this study will provide comprehensive information needed to understand the behaviour associated with overweight and obesity. This information will guide evidence-based decision-making to plan health promotion intervention programmes to address the problem of the increasing prevalence of overweight and obesity and their association to NCDs among the urban population in LMICs. Unlike in the previous studies conducted in Kenya, the findings of this study will provide information on the quantitative estimation of food intake and portion sizes as well as intensities of physical activity including sedentary time. The inclusion of qualitative data in this study will provide participants’ perceptions on the healthy lifestyle necessary to identify potential barriers to healthy living.

**References**

1. Hartung C, Lerer A, Anokwa Y, Tseng C, Brunette W, Borriello G. Open data kit: tools to build information services for developing regions. Proceedings of the 4th ACM/IEEE International Conference on Information and Communication Technologies and Development - ICTD ’10. London, United Kingdom: ACM Press; 2010. p. 1–12.
2. Kenya National Bureau of Statistics. Kenya Demographic and Health Survey 2014. Rockville, MD, USA: Kenya National Bureau of Statistics; 2015. URL: <https://www.knbs.or.ke/publications/> [accessed 2020-10-9]
3. Larios SE, Ayala GX, Arredondo EM, Baquero B, Elder JP. Development and validation of a scale to measure Latino parenting strategies related to children’s obesigenic behaviors: The Parenting strategies for Eating and Activity Scale (PEAS). Appetite 2009 Feb;52(1):166–172.
4. FAO. Direct methods. Dietary Assessment A resource guide to method selection and application in low resource settings. Rome, Italy: FAO; 2018. URL: <http://www.fao.org/3/i9940en/I9940EN.pdf> [accessed 2020-12-01]
5. Anono E, Walsh H, Kanerva N, Mubasu D, Victor O, Clinton B, Hyvönen A, Ochola S, Erkkola M, Onywera V, Fogelholm M. Photographic Food Atlas for Kenyan Pre-adolescents (9-14 years). 2019. URL: <https://kenfinedura.files.wordpress.com/2020/03/photographic-food-atlas-2-2-11-2018-1.pdf> [accessed 2020-12-03]
6. Mwai J, Kimani A, Charrondiere UR, Grande F, Rittenschober D, Vincent A, Murugu D. Kenya Food Composition Tables 2018. FAO, Government of Kenya; 2018. URL: <https://www.nutritionhealth.or.ke/programmes/healthy-diets-physical/food-composition-tables/> [accessed 2020-12-9]
7. Vila-Real C, Pimenta-Martins A, Magu J-S, Kunyanga C, Mbugua S, Katina K, Maina NH, Gomes AM, Pinto E. A culture-sensitive semi-quantitative FFQ for use among the adult population in Nairobi, Kenya: development, validity, and reproducibility. Public Health Nutr 2020 Jul 24;1–11.
8. Kennedy G, Ballard T, Dop M. Guidelines for measuring household and individual dietary diversity. Rome, Italy: FAO; 2010. ULR from: <http://www.fao.org/3/a-i1983e.pdf> [accessed 2020-12-09]
9. Nelson M, Atkinson M, Darbyshire S. Food photography. I: The perception of food portion size from photographs. Br J Nutr 1994 Nov;72(5):649–663.
10. Amougou N, Cohen E, Mbala ML, Grosdidier B, Bernard JY, Saïd-Mohamed R, Pasquet P. Development and validation of two food portion photograph books to assess dietary intake among adults and children in Central Africa. Br J Nutr 2016 Mar 14;115(5):895–902.
11. Bouchoucha M, Akrout M, Bellali H, Bouchoucha R, Tarhouni F, Mansour AB, Zouari B. Development and validation of a food photography manual, as a tool for estimation of food portion size in epidemiological dietary surveys in Tunisia. Libyan J Med 2016;11:32676.
12. Biltoft-Jensen A, Holmgaard Nielsen T, Hess Ygil K, Christensen T, Fagt S. Accuracy of food photographs for quantifying food servings in a lunch meal setting among Danish children and adults. J Hum Nutr Diet 2018;31(1):131–140.
13. Onywera VO, Larouche R, Oyeyemi AL, Prista A, Akinroye KK, Heyker S, Owino GE, Tremblay MS. Development and convergent validity of new self-administered questionnaires of active transportation in three African countries: Kenya, Mozambique and Nigeria. BMC Public Health 2018 Aug 16;18(1):1018.
14. Barreira TV, Schuna JM, Tudor-Locke C, Chaput J-P, Church TS, Fogelholm M, Hu G, Kuriyan R, Kurpad A, Lambert EV, Maher C, Maia J, Matsudo V, Olds T, Onywera V, Sarmiento OL, Standage M, Tremblay MS, Zhao P, Katzmarzyk PT. Reliability of accelerometer-determined physical activity and sedentary behavior in school-aged children: a 12-country study. Int J Obes Suppl 2015 Dec;5(Suppl 2):S29–S35.
15. Trost SG, Loprinzi PD, Moore R, Pfeiffer KA. Comparison of accelerometer cut points for predicting activity intensity in youth. Med Sci Sports Exerc 2011 Jul;43(7):1360–1368.
16. Troiano RP, Berrigan D, Dodd KW, Mâsse LC, Tilert T, McDowell M. Physical activity in the United States measured by accelerometer. Med Sci Sports Exerc 2008 Jan;40(1):181–188.
17. de Onis M, Onyango AW, Borghi E, Siyam A, Nishida C, Siekmann J. Development of a WHO growth reference for school-aged children and adolescents. Bull World Health Organ 2007 Sep;85(9):660–667.
18. R Core Team. R: A language and environment for statistical computing. Vienna, Austria.: R Foundation for Statistical Computing; 2018. URL: <https://www.R-project.org/> [accessed 2020-12-10]
19. World Food Program. Comprehensive Food Security & Vulnerability Analysis Guidelines (CFSVA 2009). Rome, Italy: World Food Program; 2009. URL: <https://documents.wfp.org/stellent/groups/public/documents/manual_guide_proced/wfp203208.pdf?_ga=2.188714593.838629093.1600947078-1715252235.1600947078> [accessed 2020-11-10]
20. Hjelm L, Miller D, Wadhwa A. VAM Guidance paper. Creation of Wealth Index. World Food Program; 2017. URL: <https://docs.wfp.org/api/documents/WFP-0000022418/download/> [accessed 2020-11-10]
21. Bernard HR, Ryan GW, Wutich A. Analyzing qualitative data: systematic approaches. 2. Ed. Sage Publications, Inc; 2016. ISBN:978-0-7619-2490-6
22. Guest G, Namey E, McKenna K. How Many Focus Groups Are Enough? Building an Evidence Base for Nonprobability Sample Sizes. Field Methods SAGE Publications Inc; 2017 Feb 1;29(1):3–22.
23. Lincoln Y, Guba E. Establishing trustworthiness. In: Bryman A & Burgess RG. Qualitative research. Sage Publications, Inc; 1999. p. 398–444.
24. Ministry of Health, Kenya National Bureau of Statistics Ministry of, WHO. Kenya Stepwise Survey for non-communicable diseases risk factors 2015 report. Nairobi, Kenya: Ministry of Health; 2015. Nairobi, Kenya; 2015. URL: <http://www.who.int/ncds/surveillance/steps/Kenya_2015_STEPS_Report.pdf> [accessed 2020-9-10]
25. Micklesfield LK, Lambert EV, Hume DJ, Chantler S, Pienaar PR, Dickie K, Puoane T, Goedecke JH. Socio-cultural, environmental and behavioural determinants of obesity in black South African women. Cardiovasc J Afr 2013 Nov;24(9–10):369–375.
