# Supplementary material for: Wealth and obesity in pre-adolescents and their guardians: A first step in explaining non-communicable disease-related behaviour in two areas of Nairobi City County
Source: PLOS Glob Public Health. 2023 Feb 28;3(2):e0000331. doi: 10.1371/journal.pgph.0000331 (PMC10021148; doi:10.1371/journal.pgph.0000331)
Supplement: S2 Text — (DOC) [file pgph.0000331.s003.doc]

**S2_Text: Additional Figures and Tables related to the construction of the Wealth Index**

S2, Fig 1: Household wealth indicators by the study areas


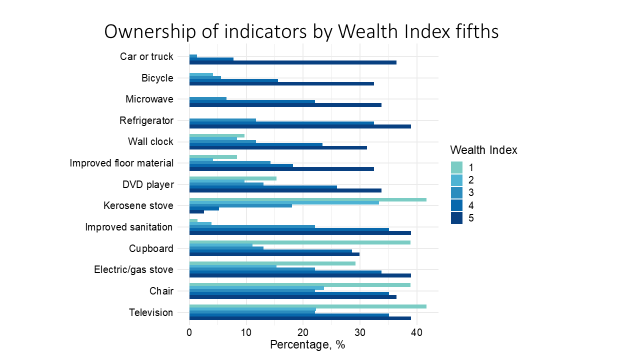


S2 Fig 2: Ownership of different indicators by wealth Index fifths (1 = lowest 20%; 5 = highest 20%).

S2 Table 1: Correlations between household wealth indicators.

|  | | Improved sanitation | Improved floor material | Television | Refrigerator | Chair | Cupboard | Wall clock | Micro-wave | DVD player | Electric or gas stove | Kerosene stove | Bicycle | Car or truck |
| --- | --- | --- | --- | --- | --- | --- | --- | --- | --- | --- | --- | --- | --- | --- |
|  | Improved sanitation | 1.00 | 0.43 | 0.31 | 0.72 | 0.32 | 0.16 | 0.48 | 0.56 | 0.39 | 0.44 | -0.67 | 0.35 | 0.44 |
|  | Improved floor material | 0.43 | 1.00 | 0.20 | 0.37 | 0.17 | 0.22 | 0.22 | 0.34 | 0.21 | 0.31 | -0.31 | 0.28 | 0.33 |
|  | Television | 0.31 | 0.20 | 1.00 | 0.31 | 0.13 | 0.35 | 0.33 | 0.25 | 0.38 | 0.40 | -0.29 | 0.23 | 0.19 |
|  | Refrigerator | 0.72 | 0.37 | 0.31 | 1.00 | 0.26 | 0.25 | 0.44 | 0.74 | 0.39 | 0.43 | -0.68 | 0.46 | 0.52 |
|  | Chair | 0.32 | 0.17 | 0.13 | 0.26 | 1.00 | 0.20 | 0.24 | 0.23 | 0.26 | 0.30 | -0.24 | 0.08 | 0.12 |
|  | Cupboard | 0.16 | 0.22 | 0.35 | 0.25 | 0.20 | 1.00 | 0.37 | 0.28 | 0.17 | 0.26 | -0.15 | 0.18 | 0.20 |
|  | Wall clock | 0.48 | 0.22 | 0.33 | 0.44 | 0.24 | 0.37 | 1.00 | 0.43 | 0.33 | 0.24 | -0.35 | 0.32 | 0.34 |
|  | Microwave | 0.56 | 0.34 | 0.25 | 0.74 | 0.23 | 0.28 | 0.43 | 1.00 | 0.44 | 0.30 | -0.47 | 0.44 | 0.48 |
|  | DVD player | 0.39 | 0.21 | 0.38 | 0.39 | 0.26 | 0.17 | 0.33 | 0.44 | 1.00 | 0.37 | -0.33 | 0.31 | 0.35 |
|  | Electric or gas stove | .440 | .305 | 0.40 | 0.43 | 0.30 | 0.26 | 0.24 | 0.30 | 0.37 | 1.00 | -0.44 | 0.31 | 0.25 |
|  | Kerosene stove | -.671 | -.308 | -0.28 | -0.68 | -0.24 | -0.15 | -0.35 | -0.47 | -0.33 | -044 | 1.00 | -0.43 | -0.50 |
|  | Bicycle | .348 | .280 | 0.23 | 0.46 | 0.08 | 0.18 | 0.32 | 0.434 | 0.31 | 0.31 | -0.43 | 1.00 | 0.47 |
|  | Car or truck | .441 | .331 | 0.19 | 0.52 | 0.12 | 0.20 | 0.34 | 0.48 | 0.35 | 0.25 | -0.50 | 0.47 | 1.00 |

S2 Table 2: KMO and Bartlett’s test results from the principal component analysis.

| Kaiser-Meyer-Olkin Measure of Sampling Adequacy | | .87 |
| --- | --- | --- |
| Bartlett's Test of Sphericity | Approx. Chi-Square | 764.068 |
|  | df | 78 |
|  | Significance | <.0001 |

S2 Table 3: Total variance explained by the household wealth indicators in the principal component analysis.

| Component | Initial Eigenvalues | | | Extraction Sums of Squared Loadings | | | Rotation Sums of Squared Loadings | | | |
| --- | --- | --- | --- | --- | --- | --- | --- | --- | --- | --- |
|  | Total | % of Variance | Cumulative % | Total | % of Variance | Cumulative % | Total | | % of Variance | Cumulative % |
| 1 | 5.246 | 40.353 | 40.353 | 5.246 | 40.353 | 40.353 | 4.067 | | 31.287 | 31.287 |
| 2 | 1.238 | 9.521 | 49.874 | 1.238 | 9.521 | 49.874 | 2.416 | | 18.587 | 49.874 |
| 3 | 0.992 | 7.631 | 57.505 |  |  |  |  | |  |  |
| 4 | 0.890 | 6.844 | 64.349 |  |  |  |  | |  |  |
| 5 | 0.838 | 6.447 | 70.796 |  |  |  |  | |  |  |
| 6 | 0.716 | 5.509 | 76.305 |  |  |  |  | |  |  |
| 7 | 0.669 | 5.150 | 81.455 |  |  |  |  | |  |  |
| 8 | 0.576 | 4.434 | 85.888 |  |  |  |  | |  |  |
| 9 | 0.536 | 4.124 | 90.012 |  |  |  |  | |  |  |
| 10 | 0.475 | 3.651 | 93.664 |  |  |  |  | |  |  |
| 11 | 0.384 | 2.956 | 96.619 |  |  |  |  | |  |  |
| 12 | 0.256 | 1.966 | 98.585 |  |  |  |  | |  |  |
| 13 | 0.184 | 1.415 | 100.000 |  |  |  |  | |  |  |
|  | | | | | | | |  | | |

S2 Table 4: Component matrix table for components 1 and 2 from the principal component analysis

| Household wealth indicators | Components | |
| --- | --- | --- |
|  | 1 | 2 |
| Refrigerator | 0.844 | -0.222 |
| Improved sanitation | 0.802 | -0.151 |
| Microwave | 0.760 | -0.181 |
| Kerosene stove | -0.750 | 0.248 |
| Car or truck | 0.654 | -0.341 |
| Wall clock | 0.615 | 0.206 |
| Electric or gas stove | 0.606 | 0.284 |
| Bicycle | 0.603 | -0.255 |
| DVD player | 0.595 | 0.169 |
| Improved floor material | 0.527 | -0.063 |
| Chair | 0.395 | 0.350 |
| Cupboard | 0.413 | 0.577 |
| Television | 0.506 | 0.527 |
