# Supplementary material for: Wealth and obesity in pre-adolescents and their guardians: A first step in explaining non-communicable disease-related behaviour in two areas of Nairobi City County
Source: PLOS Glob Public Health. 2023 Feb 28;3(2):e0000331. doi: 10.1371/journal.pgph.0000331 (PMC10021148; doi:10.1371/journal.pgph.0000331)
Supplement: S3 Text — (DOCX) [file pgph.0000331.s004.docx]

Inclusivity in global research

PLOS’ policy on inclusivity in global research aims to improve transparency in the reporting of research performed outside of researchers’ own country or community and ensures that PLOS publications reporting global research adhere to high standards for research ethics and authorship. Authors of relevant research articles may be asked to complete the questionnaire below, which outlines ethical, cultural, and scientific considerations specific to inclusivity in global research. This questionnaire may be requested when researchers have travelled to a different country to conduct research, if research uses samples collected in another country, research with Indigenous populations or their lands, or if research is on cultural artefacts. Researchers travelling to another country solely to use laboratory equipment will not normally be required to complete the questionnaire. However, the questionnaire can be requested at the journal’s discretion for any submission – if you have been requested to complete this questionnaire by the PLOS journal you submitted to, please do so.

Please complete the questionnaire below and include this as a Supporting Information file with your manuscript. Note that if your paper is accepted for publication, this checklist will be published with your article in the supporting information files. Please ensure that you reference the checklist in the main body of your manuscript. We suggest adding a subsection ‘Inclusivity in global research’ to your Methods section and adding the following sentence: “Additional information regarding the ethical, cultural, and scientific considerations specific to inclusivity in global research is included in the Supporting Information (SX Checklist)”

The questions have been designed to be applicable to a wide range of study types, and there are subsections for both human subjects research and non-human subjects research. If any of the questions are not relevant to your research please mark them as “N/A” as appropriate.

**Ethical considerations, permits and authorship**

*This section is applicable to all research types.*

Provide details as to who granted permissions and/or consent for the study to take place in the Methods section of your manuscript. This should include the names of **all** ethics boards, governmental organizations, community leaders or other bodies that provided approval for the study. If individuals provided approval refer to these people by their role or title but do not list their name(s).

Reported on page number: 233-242

If there were any deviations from the study protocol after approval was obtained please provide details of these changes in the Methods section of your manuscript.
Did this study involve local collaborators that are residents of the country where the research was conducted or members of the community studied? If you do not have any authors from said communities, please provide an explanation for this below.

Reported on page number: No deviations to be reported.

The study was a joint project between Kenyatta University (KU), Nairobi Kenya; University of Helsinki, Helsinki Finland; and Haaga-Helia University of Applied Sciences, Lahti, Finland. The study aims and design was planned together with researchers from each institution. Researchers from KU were responsible for conducting the field work. Students of KU were enumerators going to the field together with local health community volunteers, who are recognized and trusted members of the communities.

Everyone listed as an author should meet PLOS’ criteria for authorship and all individuals who meet these criteria should be included in the author byline, rather than the acknowledgements. Authorship criteria is based on the International Committee of Medical Journal Editors (ICMJE) Uniform Requirements for Manuscripts Submitted to Biomedical Journals - for further information please see here: <https://journals.plos.org/plosone/s/authorship>.

**Human subjects research (e.g. health research, medical research, cross-cultural psychology)**

Did you obtain written informed consent from a representative of the local community or region before the research took place? How did you establish who speaks for the community? Details of written informed consent obtained from study participants should be reported separately in the Methods section of your manuscript.

In addition to the official ethical clearance, we sought a clearence from the Sub-County Health Management Teams (from Embakasi and Langata) and from the local administration (chiefs) for the areas where the study was to be conducted before its commencement. We did not separately decide who speaks for the community as there is administration in place. Further, the field teams were accompanied by Community Health Volunteers who are trusted and recognized members of the communities. Eligible pre-adolescents along with their guardian(s) were given an informed consent form to sign to show willingness to participate in the study. The purpose of the study, the interviews to be done, the voluntary nature of participation, and the right to refuse to participate in any part of the study were also to be explained orally either in English or Kishwahili (whichever was participant’s preferred language). This has been explained in the Methods section of our manuscript.

How did members of the local community provide input on the aims of the research investigation, its methodology, and its anticipated outcome(s)?

The aims of the study were decided together with researchers from Kenyatta university. The sampling and locations of the study were polished together with the local teams at the health facilities with whom we were collaborating during the field work.

When engaging with the local community, how did you ensure that the informed consent documents and other materials could be understood by local stakeholders?

The field teams were all local people (students from Kenyatta University) who are fluent in local languages. The teams were accompanied by Community Health Volunteers who are recognized and trusted members of the communities. Together they made sure that the participants understood the content of the consent documents and all measures taken in the survey. They also were made clear that they can withdraw their consent at any time without experiencing any consequences or need to explain why they want to abort. All questionnaires were available in English and Kiswahili and the enumerators were also fluent in both languages.

Will the findings of the research be made available in an understandable format to stakeholders in the community where the study was conducted (e.g. via a presentation, summary report, copies of publications, etc.)? Please provide details of how this will be achieved.

The research results have been shared with the stakeholders in the communities in a local seminar, arranged in November 2019. Further, any publications will be made available through open publishing to the local stakeholders, mainly the sub-county health management teams. The project (KENFIN-EDURA) has a webpage where all publications / links to publications are gathered for easy access of the results.

**Non-human subjects research using specimens/ animals collected as part of the study, or those housed in archival collections. Examples include archaeology, paleontology, botany and zoology.**

Did the permission you obtained from a local authority to perform the study include an agreement on access to outputs and benefit sharing? This may include procedures to enable fair distribution of the benefits and resources arising from the research performed. Please include any details of Prior Informed Consent and Benefit Sharing Agreements obtained. These may be required by field-specific regulations, for example the Convention on Biological Diversity (CBD) and the associated Nagoya Protocol.

NOT APPLICABLE

If the material used in your study was imported, please A) provide the year it was imported and B) indicate whether permits were obtained to import/export the materials used, C) provide details of any permits obtained. If this information is not available, please indicate this.

NOT APPLICABLE

If you used archival specimens, please state how the material used in your study was acquired by the institute it is held in and provide details of any permits obtained for the original excavations/ sample collection. If this information is not available, please indicate this.

NOT APPLICABLE

How was the potential cultural significance of the materials collected in your study to local communities considered in your research design? Were Indigenous peoples and/or local researchers and institutions involved with archaeological excavations / collection of specimens? If so, please provide a description of their involvement.

NOT APPLICABLE

If your manuscript includes photographs of human remains please indicate whether authors obtained permission from descendants or affiliated cultural communities to do so.

NOT APPLCABLE
