# Supplementary material for: Wealth and obesity in pre-adolescents and their guardians: A first step in explaining non-communicable disease-related behaviour in two areas of Nairobi City County
Source: PLOS Glob Public Health. 2023 Feb 28;3(2):e0000331. doi: 10.1371/journal.pgph.0000331 (PMC10021148; doi:10.1371/journal.pgph.0000331)
Supplement: Attachment — Submitted filename: RESPONSE TO REVIEWERS.docx [file pgph.0000331.s005.docx]

Dear Dr. Ochola,

Thank you for submitting your manuscript to PLOS Global Public Health. After careful consideration, we feel that it has merit but does not fully meet PLOS Global Public Health’s publication criteria as it currently stands. Therefore, we invite you to submit a revised version of the manuscript that addresses the points raised during the review process.

Please submit your revised manuscript by 15/09/2022. If you will need more time than this to complete your revisions, please reply to this message or contact the journal office at [globalpubhealth@plos.org](mailto:globalpubhealth@plos.org). When you're ready to submit your revision, log on to <https://www.editorialmanager.com/pgph/> and select the 'Submissions Needing Revision' folder to locate your manuscript file.

Please include the following items when submitting your revised manuscript:

- A rebuttal letter that responds to each point raised by the editor and reviewer(s). You should upload this letter as a separate file labeled 'Response to Reviewers'.
- A marked-up copy of your manuscript that highlights changes made to the original version. You should upload this as a separate file labeled 'Revised Manuscript with Track Changes'.
- An unmarked version of your revised paper without tracked changes. You should upload this as a separate file labeled 'Manuscript'.

Guidelines for resubmitting your figure files are available below the reviewer comments at the end of this letter.

We look forward to receiving your revised manuscript.

Kind regards,

Catherine Elizabeth Draper
Academic Editor
PLOS Global Public Health

Journal Requirements:

1. Please include a complete copy of PLOS’ questionnaire on inclusivity in global research in your revised manuscript. Our policy for research in this area aims to improve transparency in the reporting of research performed outside of researchers’ own country or community. The policy applies to researchers who have travelled to a different country to conduct research, research with Indigenous populations or their lands, and research on cultural artefacts. The questionnaire can also be requested at the journal’s discretion for any other submissions, even if these conditions are not met.  Please find more information on the policy and a link to download a blank copy of the questionnaire here: <https://journals.plos.org/plosone/s/best-practices-in-research-reporting>. Please upload a completed version of your questionnaire as Supporting Information when you resubmit your manuscript.”

*Authors: We have completed the questionnaire and submitted it with our revised manuscript.*

2. We ask that a manuscript source file is provided at Revision. Please upload your manuscript file as a .doc, .docx, .rtf or if latex provide both .pdf and .tex.

*Authors: The manuscript file is uploaded as word document.*

3. Please amend your detailed Financial Disclosure statement. This is published with the article. It must therefore be completed in full sentences and contain the exact wording you wish to be published.

a. Please clarify all sources of funding (financial or material support) for your study. List the grants (with grant number) or organizations (with url) that supported your study, including funding received from your institution.

*Authors: This has been amended in the Manuscript (line 403) and submission:*

*“This study was part of a collaborative project “The Kenya-Finland Education and Research Alliance (KENFIN-EDURA)” (state grant HEL7M0453-82, sum 527,000 EUR awarded to MF and VO) funded by the Finnish Ministry of Foreign Affairs through The Higher Education Institutions Institutional Cooperation Instrument (HEI ICI; https://www.oph.fi/en/programmes/hei-ici-programme). The funders had no role in study design, data collection and analysis, decision to publish, or preparation of the manuscript.”*

b. State the initials, alongside each funding source, of each author to receive each grant.

*Authors: This has been amended in the Manuscript and submission. See above.*

c. State what role the funders took in the study. If the funders had no role in your study, please state: “The funders had no role in study design, data collection and analysis, decision to publish, or preparation of the manuscript.”

*Authors: This has been amended in the Manuscript and submission. See above.*

4. Please update the 'Competing Interests' statement with this "The authors have declared that no competing interests exist".

*This has been done in the Manuscript and submission.*

5. Please provide separate figure files in .tif or .eps format.

*Authors: the figures have been converted to tiff.*

For more information about figure files please see our guidelines:

<https://journals.plos.org/globalpublichealth/s/figures> 
<https://journals.plos.org/globalpublichealth/s/figures#loc-file-requirements>

6. We have noticed that you have uploaded Supporting Information files, but you have not included a list of legends. Please add a full list of legends for your Supporting Information files after the references list.

*Full list of legends for the supporting Information included after the references list*

7. In the online submission form, you indicated that "THE DATA IS AVAILABLE BY REQUEST FROM HELSINKI UNIVERSITY FINLAND OR KENYATTA UNIVERSITY, NAIROBI KENYA". All PLOS journals now require all data underlying the findings described in their manuscript to be freely available to other researchers, either 1. In a public repository, 2. Within the manuscript itself, or 3. Uploaded as supplementary information.

This policy applies to all data except where public deposition would breach compliance with the protocol approved by your research ethics board. If your data cannot be made publicly available for ethical or legal reasons (e.g., public availability would compromise patient privacy), please explain your reasons by return email and your exemption request will be escalated to the editor for approval. Your exemption request will be handled independently and will not hold up the peer review process, but will need to be resolved should your manuscript be accepted for publication. One of the Editorial team will then be in touch if there are any issues.

*Authors’ reply: This is not a problem. We will upload all relevant data in an autonymized form to Dryad* [*https://datadryad.org/stash*](https://datadryad.org/stash)*. This will be done no later than 15 Sept 2022. We will send you the precise www address immediately after this has been done.*

Additional Editor Comments (if provided):


[Note: HTML markup is below. Please do not edit.]

Reviewers' comments:

Reviewer's Responses to Questions

**Comments to the Author**

1. Does this manuscript meet PLOS Global Public Health’s [publication criteria](https://journals.plos.org/globalpublichealth/s/criteria-for-publication)? Is the manuscript technically sound, and do the data support the conclusions? The manuscript must describe methodologically and ethically rigorous research with conclusions that are appropriately drawn based on the data presented.

Reviewer #1: Yes

2. Has the statistical analysis been performed appropriately and rigorously?

Reviewer #1: Yes

3. Have the authors made all data underlying the findings in their manuscript fully available (please refer to the Data Availability Statement at the start of the manuscript PDF file)?

The [PLOS Data policy](https://journals.plos.org/globalpublichealth/s/data-availability) requires authors to make all data underlying the findings described in their manuscript fully available without restriction, with rare exception. The data should be provided as part of the manuscript or its supporting information, or deposited to a public repository. For example, in addition to summary statistics, the data points behind means, medians and variance measures should be available. If there are restrictions on publicly sharing data—e.g. participant privacy or use of data from a third party—those must be specified.

Reviewer #1: Yes

4. Is the manuscript presented in an intelligible fashion and written in standard English?

PLOS Global Public Health does not copyedit accepted manuscripts, so the language in submitted articles must be clear, correct, and unambiguous. Any typographical or grammatical errors should be corrected at revision, so please note any specific errors here.

Reviewer #1: Yes

5. Review Comments to the Author

Please use the space provided to explain your answers to the questions above. You may also include additional comments for the author, including concerns about dual publication, research ethics, or publication ethics. (Please upload your review as an attachment if it exceeds 20,000 characters)

Reviewer #1: This study examines associations between wealth, income, and bmi z-scores in pre-adolescents and found that higher wealth and income predicted higher bmi z-scores. The study's hypotheses are grounded in the literature and are in line with other studies in similar countries.

Several times throughout the paper, the phrase "results suggest a rapid transition in lifestyle...". I suggest avoiding the word "transition" because it sounds like the study examined changes in patterns over time instead of associations. I think the authors should reword this.

*Authors: This is a very good point. We have made changes in all points referring to our paper/result but kept “transition” when it appeared in a more general context.*

Lines 60-61 ("The overall aim of this study..."): Remove the word "interactions". This led me to believe the study examined statistical interactions, but it did not.

*Authors: Thank you for the comment, change done.*

Lines 62-64: Do not include the purpose of the qualitative portion here. It sounds like this paper includes the qualitative results. Consider including this information in the "future directions" paragraph in the discussion.

*Authors: Good point. The part describing the qualitative study was removed and paragraph slightly changed.*

Lines 148-150: Do not need to include how interviews were conducted.

*Authors: We have removed the referral to the qualitative part.*

Lines 177: Include version of R software.

*Authors: we used the version 3.6.3. This has been added to the manuscript.*

Lines 189-196: Move this information to the measures section. Information about the measure does not belong in the statistical analysis section.

*Authors: The challenge in the description of the creation of the wealth index is that part of could fit the general Methods, as You noted. Thus, we moved the part that we thought suits to the Methods section under sub-heading “Background data” (lines 170-176):*

“Wealth is often measured in terms of economic status and living standards of households. However, the income, expenditure and consumption data needed for calculating these can be challenging to measure accurately. For this reason, we chose to use the Wealth Index similarly to the Demographic and Health Surveys (DHS) [8] and World Food Programme (WFP) Surveys [19]. The calculation of the Index is explained in the section of statistical analyses.”

*The other part of the text is purely a statistical description of the principal component analysis, and the iteration rounds before the final index was created. Thus, those parts we left in the Statistical Analysis section.*

Statistical analysis section: Clarify how your linear and logistic regression models were set up (i.e., outcome variable, predictors, covariates).

*Authors: these have been now clarified in the Statistical Analysis section as follows (lines 203-212):*

“Difference in anthropometric measurements between study areas was analyzed using analysis of variance or logistic regression. The continuous anthropometric measures (BMI, z-BMI score, MUAC and waist circumference) were treated as outcome variables and a binary variable indicating study area was used as independent variable in the analysis of variance. In the logistic regression, the outcome variables were binary variables for underweight, overweight, obesity, MUAC, and central obesity. All analyses were adjusted of participants’ sex and age. Further, we analyzed the associations of wealth (categorical independent variable) and income (categorical independent variable) with BMI and BMI z-score (continuous outcome variables) by using a linear regression model, adjusted for age and sex. The same analyses with obesity and overweight as binary outcome variables were done by using a logistic regression model.”

*Further, we noticed that we had not explained properly the binary anthropometric variables, thus we added a description of those in the Methods section (lines 190-196):*

“For pre-adolescents, underweight was defined as BMI z-score < -2.0, overweight as BMI z-score >1.0 and obesity as BMI z-score >2.0. For thinness, we used MUAC values <18.5 cm and <16.0 cm. For adult underweight, overweight and obesity, we used international cut-offs of BMI <18.5 kg/m^2^, >24.9 kg/m^2^, and >29.9 kg/m^2^. Waist/abdominal obesity was defined as >88.0 cm for women and >102 cm for men. For adults, MUAC cut-offs for underweight were defined as <22.0 cm for women and <23.cm for men.”

Results - please provide a table displaying all regression model results.

*Authors: We are willing to modify the tables, but it is unclear to us, to which regression models You are referring to. Table 2 includes P-values both from analysis of variance AND logistic regression. We feel that including both beta coefficients and standard errors (logistic regression) plus sum of squares and mean square (ANOVA) into this table would make it difficult for readers to follow and therefore we are unsure if this is really the table that You are referring to. Supplement 3, tables 1 and 2 include results from linear and logistic regression and there we have already provided beta coefficients and standard errors in addition to P-values. Thus, we are not sure what could be added in this table from the regression modes. Perhaps we could include values for the confounding factors used in the model (age and sex)?*

Results - include if there were significant demographic differences between included and excluded participants

*Authors: In the sampling phase, families who fulfilled the study criteria were mapped both study areas by the Community Health Volunteers. These families where then approached by the Volunteers to seek preliminary willingness to participate the study. There were only few families (8 in Embakasi and 3 in Langata) that did not want to take part and no data was collected from them. The other families who were willing to participate were then visited by the data collection team who explained the study, collected informed consents and after that took the measurements. Therefore, the families that did not fulfill the study criteria and thus were excluded from the sample, we do not have any information on.The vast majority of the families invited participated in the study. This gives us a reason to expect that the families that fulfilled our study criteria and were included do not differ from those who were not included. We have added this explanation to the Discussion section, in the part where strengths and limitations are discussed (lines 401-405).*

*Further, collecting the data in electronical format using Open Data Kit allowed to control that we did not have any missing data for any of the variables used in the analysis of this study. Therefore, we did not have to exclude any participants from the analysis presented in our manuscript.*

Results - a beta of .01 is very small. This should be acknowledged as a limitation in the discussion and its practical significance should be discussed.

*Authors: You are right, a sentence has been added to the discussion (weaknesses of the study):*

Moreover, in the linear regression model, the beta-coefficient for Wealth Index against BMI was very small (0.01), albeit significant. Hence, the interpretation should be done with caution.

Lines 290-291: Provide statistics to support this sentence.

*Authors: We added (line 333-335):”… the highest or two highest classes had an OR statistically significantly different from reference, see Supplement 3, Table 1),…”*

6. PLOS authors have the option to publish the peer review history of their article ([what does this mean?](https://journals.plos.org/globalpublichealth/s/editorial-and-peer-review-process#loc-peer-review-history)). If published, this will include your full peer review and any attached files.


**Do you want your identity to be public for this peer review?** If you choose “no”, your identity will remain anonymous but your review may still be made public.


For information about this choice, including consent withdrawal, please see our [Privacy Policy](https://www.plos.org/privacy-policy).

Reviewer #1: No

[NOTE: If reviewer comments were submitted as an attachment file, they will be attached to this email and accessible via the submission site. Please log into your account, locate the manuscript record, and check for the action link "View Attachments". If this link does not appear, there are no attachment files.]

While revising your submission, please upload your figure files to the Preflight Analysis and Conversion Engine (PACE) digital diagnostic tool, <https://pacev2.apexcovantage.com/>. PACE helps ensure that figures meet PLOS requirements. To use PACE, you must first register as a user. Registration is free. Then, login and navigate to the UPLOAD tab, where you will find detailed instructions on how to use the tool. If you encounter any issues or have any questions when using PACE, please email PLOS at [figures@plos.org](mailto:figures@plos.org). Please note that Supporting Information files do not need this step.

*In compliance with data protection regulations, you may request that we remove your personal registration details at any time.* [*(Remove my information/details)*](https://www.editorialmanager.com/pgph/login.asp?a=r)*. Please contact the publication office if you have any questions.*
