# Supplementary material for: Wealth and obesity in pre-adolescents and their guardians: A first step in explaining non-communicable disease-related behaviour in two areas of Nairobi City County
Source: PLOS Glob Public Health. 2023 Feb 28;3(2):e0000331. doi: 10.1371/journal.pgph.0000331 (PMC10021148; doi:10.1371/journal.pgph.0000331)
Supplement: Attachment — Submitted filename: RESPONSE TO REVIEWERS.docx [file pgph.0000331.s006.docx]

PLOS Global Public Health Decision: Revision required [PGPH-D-22-00151R1] - [EMID:fabe08e7b053fd19]

External

Inbox

| **PLOS Global Public Health <em@editorialmanager.com>** | Thu, 8 Dec 2022, 04:14 |  |    |
| --- | --- | --- | --- |
| to Sophie  | | |  |

CC: [catherine.draper@wits.ac.za](mailto:catherine.draper@wits.ac.za)

PGPH-D-22-00151R1
WEALTH AND OBESITY IN PRE-ADOLESCENTS AND THEIR GUARDIANS: A FIRST STEP IN EXPLAINING NON-COMMUNICABLE DISEASE-RELATED BEHAVIOUR IN TWO AREAS OF NAIROBI CITY COUNTY
PLOS Global Public Health

Dear Dr. Ochola,

Thank you for submitting your manuscript to PLOS Global Public Health. After careful consideration, we feel that it has merit but does not fully meet PLOS Global Public Health’s publication criteria as it currently stands. Therefore, we invite you to submit a revised version of the manuscript that addresses the points raised during the review process.

Please submit your revised manuscript by Jan 21 2023 11:59PM. If you will need more time than this to complete your revisions, please reply to this message or contact the journal office at [globalpubhealth@plos.org](mailto:globalpubhealth@plos.org). When you're ready to submit your revision, log on to <https://www.editorialmanager.com/pgph/> and select the 'Submissions Needing Revision' folder to locate your manuscript file.

Please include the following items when submitting your revised manuscript:

- A rebuttal letter that responds to each point raised by the editor and reviewer(s). You should upload this letter as a separate file labeled 'Response to Reviewers'.
- A marked-up copy of your manuscript that highlights changes made to the original version. You should upload this as a separate file labeled 'Revised Manuscript with Track Changes'.
- An unmarked version of your revised paper without tracked changes. You should upload this as a separate file labeled 'Manuscript'.

Guidelines for resubmitting your figure files are available below the reviewer comments at the end of this letter.

We look forward to receiving your revised manuscript.

Kind regards,

Catherine Draper
Academic Editor
PLOS Global Public Health

Journal Requirements:

1. We noticed that you used "not shown" in the manuscript. We do not allow these references, as the PLOS data access policy requires that all data be either published with the manuscript or made available in a publicly accessible database. Please amend the supplementary material to include the referenced data or remove the references.

Additional Editor Comments (if provided):

The reviewer had some further comments that need addressing before the manuscript can be considered for publication.


[Note: HTML markup is below. Please do not edit.]

Reviewers' comments:

Reviewer's Responses to Questions

**Comments to the Author**

1. If the authors have adequately addressed your comments raised in a previous round of review and you feel that this manuscript is now acceptable for publication, you may indicate that here to bypass the “Comments to the Author” section, enter your conflict of interest statement in the “Confidential to Editor” section, and submit your "Accept" recommendation.

Reviewer #1: (No Response)

2. Does this manuscript meet PLOS Global Public Health’s [publication criteria](https://journals.plos.org/globalpublichealth/s/criteria-for-publication)? Is the manuscript technically sound, and do the data support the conclusions? The manuscript must describe methodologically and ethically rigorous research with conclusions that are appropriately drawn based on the data presented.

Reviewer #1: Yes

3. Has the statistical analysis been performed appropriately and rigorously?

Reviewer #1: Yes

4. Have the authors made all data underlying the findings in their manuscript fully available (please refer to the Data Availability Statement at the start of the manuscript PDF file)?

The [PLOS Data policy](https://journals.plos.org/globalpublichealth/s/data-availability) requires authors to make all data underlying the findings described in their manuscript fully available without restriction, with rare exception. The data should be provided as part of the manuscript or its supporting information, or deposited to a public repository. For example, in addition to summary statistics, the data points behind means, medians and variance measures should be available. If there are restrictions on publicly sharing data—e.g. participant privacy or use of data from a third party—those must be specified.

Reviewer #1: Yes

5. Is the manuscript presented in an intelligible fashion and written in standard English?

PLOS Global Public Health does not copyedit accepted manuscripts, so the language in submitted articles must be clear, correct, and unambiguous. Any typographical or grammatical errors should be corrected at revision, so please note any specific errors here.

Reviewer #1: Yes

6. Review Comments to the Author

Please use the space provided to explain your answers to the questions above. You may also include additional comments for the author, including concerns about dual publication, research ethics, or publication ethics. (Please upload your review as an attachment if it exceeds 20,000 characters)

Reviewer #1: The lines listed in the response to review did not match the manuscript, so it was difficult to find the changes that were made.

Lines 60-61: The word "interactions" was not removed. I suggest changing the purpose statement to something like: "The overall aim of this study was to examine indicators of wealth and obesity (i.e., poverty, education, gender, and place of residence) in association with physical (in)activity, dietary quality and variability, and overweight/obesity among guardians and pre-adolescent children in low- and middle-income areas in urban Nairobi.

**The Word interaction deleted and the parenthesis revised**

Lines 62-62: The part describing the qualitative study was not removed as stated in the response to review.

**The statement has been deleted**

Lines 78: There is only one parenthesis in this sentence: "In this paper, we report methods and results from the socio-demographic indices, including wealth and anthropometric assessments)."

**Parenthesis merged with the previous one**

Lines 137: Consider renaming this section. Instead of "Background data" maybe call it something like "Socioeconomic data", "Wealth indicator data" or "Survey data". "Background data" is very vague and I wasn't sure what it was referring to.

**Has been renamed to read Socio-demographic characteristics**

Lines 149-154: Thank you for moving information about the Wealth Index to this paragraph. I think it fits well in this section.

**Thanks**

Lines 181-190: Thank you for including more information on how the linear and logistic regression models were set up. Could you also include how each binary variable was coded? For example, overweight/obese=1; not overweight/obese=0?

**Included**

Lines 290-292: Consider removing the sentence: "As could be expected from the comparison of the two sub-counties, both wealth (quintile classification) and income (class) predicted higher BMI z-score in adolescents." A sentiment like this should be in the discussion where you state what you expected or did not expect to find. I suggest only reporting the results in this section.

**Statement deleted**

Line 316: Consider removing "e.g.," from this sentence so that it reads: "...although the rather limited number of households prevents us from an accurate estimation of prevalence."

**Removed and sentence edited**

Line 314: Consider removing "It is expected that the..." from the beginning of the sentence in line 314 so that the sentence reads: "The findings of this research will provide novel and important new data on obesity..."

**Removed and sentence edited**


Lines 359: I think you are referring to "sub-counties" not "sub-countries".

**Corrected**

Lines 304-310 (first paragraph of discussion): Consider changing this paragraph to explain the overall takeaways from the paper before discussing specific findings.

**Corrected**


Lines 324-329: This information should go in the limitations/weaknesses paragraph.

**Corrected**


Discussion: I suggest adding a paragraph on discussing the finding that wealth and income predicted higher BMI z-scores in pre-adolescents. This is one of the most important findings.

**DONE - see lines 355 TO 358**

7. PLOS authors have the option to publish the peer review history of their article ([what does this mean?](https://journals.plos.org/globalpublichealth/s/editorial-and-peer-review-process#loc-peer-review-history)). If published, this will include your full peer review and any attached files.

**DO NOT PUBLISH
Do you want your identity to be public for this peer review?** If you choose “no”, your identity will remain anonymous but your review may still be made public.


For information about this choice, including consent withdrawal, please see our [Privacy Policy](https://www.plos.org/privacy-policy).

Reviewer #1: No

[NOTE: If reviewer comments were submitted as an attachment file, they will be attached to this email and accessible via the submission site. Please log into your account, locate the manuscript record, and check for the action link "View Attachments". If this link does not appear, there are no attachment files.]

While revising your submission, please upload your figure files to the Preflight Analysis and Conversion Engine (PACE) digital diagnostic tool, <https://pacev2.apexcovantage.com/>. PACE helps ensure that figures meet PLOS requirements. To use PACE, you must first register as a user. Registration is free. Then, login and navigate to the UPLOAD tab, where you will find detailed instructions on how to use the tool. If you encounter any issues or have any questions when using PACE, please email PLOS at [figures@plos.org](mailto:figures@plos.org). Please note that Supporting Information files do not need this step.
